# Photoinduced Absorption Spectroscopy of Photoelectrocatalytic Methylene Blue Oxidation on Titania and Hematite: The Thermodynamic and Kinetic Impacts on Reaction Pathways

**DOI:** 10.1002/advs.202206685

**Published:** 2023-01-22

**Authors:** Xinyue Guo, Zixuan Ma, Yuling Yuan, Yan Kang, Hong Xu, Zhiping Mao, Yimeng Ma

**Affiliations:** ^1^ Key Laboratory of Science and Technology of Eco‐Textile, Ministry of Education College of Chemistry and Chemical Engineering Donghua University Shanghai 201620 China; ^2^ National Innovation Center of Advanced Dyeing & Finishing Technology Shandong Zhongkang Guochuang Research Institute of Advanced Dyeing & Finishing Technology Co., Ltd. Taian City Shandong Province 271000 China; ^3^ Shanghai Jahwa United Co., Ltd. Shanghai 200082 China

**Keywords:** hematite, mineralization, photoinduced absorption spectroscopy, titania, valence band potential

## Abstract

Photoelectrochemical oxidation of methylene blue is investigated, with particular focus on the difference in kinetics and thermodynamics of decoloration and mineralization employing photoinduced absorption spectroscopy. Hematite and titania photoanodes are used for the comparison of both reactions, which is determined to be associated with the depth of the valence band (3.2 vs 2.5 V for titania and hematite, respectively). Methylene blue is mineralized by the titania photoanode, however it is only oxidized to small fragments by hematite. Such difference is related to the valence band potential that provides the thermodynamic driving force for photogenerated holes in both materials. In addition, the kinetic competition of water oxidation is found to occur on titania by controlling the pH of the electrolyte. In the pH 14 electrolyte, mineralization of methylene blue is suppressed due to the faster and dominant kinetics of water oxidation, in contrast to the complete mineralization in the near neutral electrolyte where water oxidation kinetics are modest. These results clearly address the importance considering both thermodynamic and kinetic challenges of methylene blue oxidation, which has been thought to be an easy molecule to oxidize, as the model reaction in the application of photo(electro)catalysis using metal oxides.

## Graphical abstract

Photoinduced absorption spectroscopy is employed to distinguish decoloration and mineralization of methylene blue using titania and hematite photoanodes. The valence band potential is found to be directly related to oxidizability of the organic molecule. Titania can oxidize the methylene blue to CO_2_ by deep valence band potential, whereas the oxidation stops at catechol by hematite modest valence band potential.

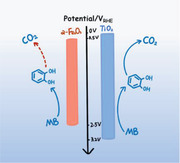

## 1 Introduction

Photoelectrochemical (PEC) oxidation reaction using semiconducting photoelectrodes is central for the application of solar energy conversion and storage.^[^ ^1^ ^]^ The oxidation involves using the photogenerated holes to react with oxidation substrates. Typical oxidations include water oxidation to form oxygen^[^ ^2^ ^]^ or hydrogen peroxide,^[^ ^3^ ^]^ organic oxidation with high selectivity^[^ ^4^ ^]^ or complete alcohol oxidation to CO_2_ for alcohol‐hydrogen reforming,^[^ ^5^ ^]^ bio‐mass and plastic reforming to produce hydrogen^[^ ^6^ ^]^ and mineralization of organic pollutants or toxins for water purification.^[^ ^7^ ^]^ The reaction pathway therefore has to be precisely controlled according to each specific reaction. For example, the PEC alcohol oxidation requires the termination of oxidation process at aldehyde formation, thus avoiding further oxidation to acid or CO_2_ by the photogenerated holes. On the other hand, the decontamination of water specifically needs to oxidize all organic molecules to CO_2_ regardless the intermediate formed during the oxidation. These two types of reactions illustrate the importance of considering the reaction pathway for the desired oxidation product via PEC oxidation.

Amongst the above‐mentioned applications, the mineralization of organic substances has been used in the application of environmental photocatalysis. It is often coupled with hydrogen production technique to achieve both water purification and hydrogen production within one system. The mineralization requires complete oxidation of organic molecules to inorganic carbon (e.g., CO_2_ or carbonate) by the photogenerated holes. The oxidation process involves a first‐step oxidation to break down the organic molecule to small organic fragments. This first‐step reaction has been often applied in the study of water purifications using photocatalysis or photoelectrocatalysis as the indication of reaction process characterized by UV–vis spectroscopy or chromatography.^[^ ^7^, ^8^ ^]^ On the other hand, mineralization requires further oxidation of these small molecules to inorganic carbon, where the detection of total organic carbon (TOC) or chemical oxygen demand (COD) is more meaningful. However, the small molecules only indicate that the structure of toxins or pollutants has been partially decomposed. The potential hazard still remains to the environment, which requires the further oxidation to CO_2_. Such process, however, has not been significantly considered to date.

One of the challenges in mineralization is often related to the n‐type semiconducting materials where oxidations take place. Semiconductors with the property of visible light absorption have been widely used for both photocatalysts and photoelectrodes. These materials often consist of the valence band potential between 1.5‐2.5 V_RHE_ (versus the reversible hydrogen electrode, RHE) with the absorption edge ≈500–700 nm. In addition, most of these materials have shown their excellent water oxidation efficiency, giving rise to the potential of organic oxidation with higher thermodynamic driving force than water oxidation (1.23 V_RHE_). The oxidation of organic molecules, for example methylene blue (MB) by bismuth vanadate (BiVO_4_) or hematite (*α*‐Fe_2_O_3_), has been reported with 100% decoloration efficiency.^[^ ^9^ ^]^ However, the secondary oxidation of the decolorated small molecules has not been frequently reported or reported with modest ability of mineralization (≈50% of TOC removal).^[^ ^10^ ^]^ Similar results have also been observed using carbon nitride (g‐C_3_N_4_),^[^ ^11^ ^]^ bismuth oxobromide (BiOBr)^[^ ^12^ ^]^ or bismuth oxide (Bi_2_O_3_),^[^ ^13^ ^]^ further demonstrating the challenge in complete oxidation of organic molecules.

In contrast to the visible light absorbing materials, semiconductors with UV light absorption have illustrated remarkable abilities for organic mineralization. Titania (TiO_2_) is a typical n‐type semiconductor that has been reported with 97–100% TOC removal of persistent pollutants (4‐chlorophenol, 4‐CP, or bisphenol A, BPA).^[^ ^7^, ^14^ ^]^ The oxidation potential of the photogenerated holes is ≈3.2 V_RHE_ due to the deeper valence band in TiO_2_, significantly larger than the visible light absorbing materials ≈1.5‐2.5 V_RHE_. Such strong oxidation power suggests a potential advantage in the mineralization of organic molecules. Pervious water oxidation studies indicated that the deep valence band potential is able to greatly enhance the water oxidation kinetics by the higher thermodynamic driving force between the valence band potential of TiO_2_ and oxygen evolution redox potential (1.23 V_RHE_).^[^ ^15^ ^]^ In addition, several studies have reported that TiO_2_ has lower selectivity in PEC oxidation of organic synthesis than *α*‐Fe_2_O_3_, including alcohol oxidation to aldehyde^[^ ^16^ ^]^ and methyl phenyl sulfide (MPS) oxidation to methyl phenyl sulfoxide (MPSO),^[^ ^4c^ ^]^ suggesting that secondary or further oxidation is likely to occur on TiO_2_, opposite to the results employing *α*‐Fe_2_O_3_. Mechanistic investigations have been carried out to uncover the underlying phenomenon between decolaration and mineralization. Active oxygen or hydroxyl radicals as the intermediate of water oxidation have been proposed to oxidize organic molecules.^[^ ^17^ ^]^ However, the oxidation by radicals suggest that the oxidation of organic molecules should be independent of photocatalysts because the same type of radicals must have identical redox potential to the targeted organic molecule, thus having the invariant oxidation power for organic oxidation. The difference in TOC removal, on the other hand, strongly suggests that there is a clear difference in the oxidation power, likely dependent on different semiconductors. Therefore, the inherent mechanisms, especially including the identification of the active species for organic oxidation, the correlation between the thermodynamic driving force and ability of mineralization, and the kinetic competition between water oxidation and organic oxidation, have not been greatly discussed, which is the focus of this study reported herein.

This study is primarily focused upon revealing the underlying mechanism of PEC organic oxidation using TiO_2_, in comparison with *α*‐Fe_2_O_3_. Methylene blue was used as the indication of decoloration and mineralization. Although MB has been used commonly in photocatalysis of dye degradation mimicking water purification, and considered as an easy dye to decompose, the mineralization shows significant difficulties with certain conditions that must be considered, giving it a perfect model system to demonstrate the challenges in organic mineralization. Operando photoinduced absorption (PIA) spectroscopy was employed to investigate the charge carrier dynamics in TiO_2_ and *α*‐Fe_2_O_3_ photoanodes for MB oxidation in decolaration and mineralization. Correlation between the reaction kinetics and their valence band potential was established to further illustrate the limitation of mineralization in the semiconductors with shallow valence band potential, and to provide the modification strategies for both materials to achieve 100% mineralization capability.

## 2 Results

This study primarily focuses upon the selectivity of MB oxidation between decoloration and mineralization on TiO_2_ and *α*‐Fe_2_O_3_. Despite broad interests in the MB photocatalytic oxidation, the concentration of MB in the electrolytes has not been considered to affect the reaction pathway. One of the significant challenges for mineralization is the low concentration of organic substrate where water oxidation becomes kinetically competitive. Therefore, the low concentration of MB employed in this study is to distinguish whether or not mineralization of MB is able to take place. Such phenomenon is thought to be more meaningful for real application in complete removal of organic compounds in water purification. In this study, low concentration (5 ppm) of MB was used to investigate the oxidation pathway with kinetic competition with water oxidation.

The TiO_2_ and *α*‐Fe_2_O_3_ photoanodes were fabricated on fluorine‐doped tin oxide (FTO) using a modified metal‐organic deposition method from the previous report of BiVO_4_. X‐ray diffraction results indicate that both TiO_2_ and *α*‐Fe_2_O_3_ are polycrystalline shown in Figure S1 (Supporting Information). Both electrodes, shown in scanning electron microscopy in Figure S2 (Supporting Information), compressed a dense and flat surface with the thickness of 1.2 µm and 500 nm for TiO_2_ and *α*‐Fe_2_O_3_, respectively.

**Figure 1:**
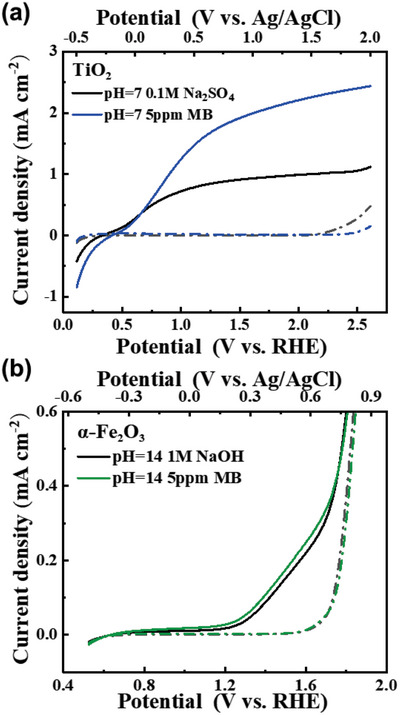
Current density–potential response of the a) TiO_2_ and b) *α*‐Fe_2_O_3_ photoanodes measured under the illumination of a 365 nm LED for water oxidation and methylene blue oxidation. Scan rate: 10 mV s^−1^. Excitation intensity: 8.8 mW cm^−2^.

**Figure** **1** shows the current density‐potential (J‐V) response of TiO_2_ and *α*‐Fe_2_O_3_ photoanodes measured under 365 nm illumination. It is clear that both photoanodes show an enhanced photocurrent in the presence of a trace amount of MB (5 ppm), suggesting that MB is more in favor of being oxidized on *α*‐Fe_2_O_3_. We note that the photocurrent of MB oxidation measured in the *α*‐Fe_2_O_3_ photoanode is marginally higher than that of water oxidation. In order to confirm this, oxygen and hydrogen evolution during bulk PEC MB oxidation was carried out and the faradaic efficiency (FE) was calculated shown in Figure S3a–c (Supporting Information). Both *α*‐Fe_2_O_3_ and TiO_2_ photoanodes show 100% FE for H_2_ evolution and 0% for O_2_ evolution, strongly suggesting that MB oxidation is kinetically competitive even at low concentration against water oxidation on the surface of these two materials. However, it is noted that the MB oxidation observed in the PEC measurements is indicative of a decoloration process, where the chemical structure of MB is decomposed during the PEC oxidation. Such oxidation is thermodynamically more favorable than water oxidation at 1.23 V_RHE_, consistent with general organic oxidation with greater thermodynamic driving force using metal oxide photoanodes.^[^ ^4^, ^7^, ^9^, ^10^, ^18^ ^]^ However, it is yet unclear whether this decomposition results in the MB structure to be oxidized to small organic molecules or inorganic carbon, such as CO_2_.

**Figure 2:**
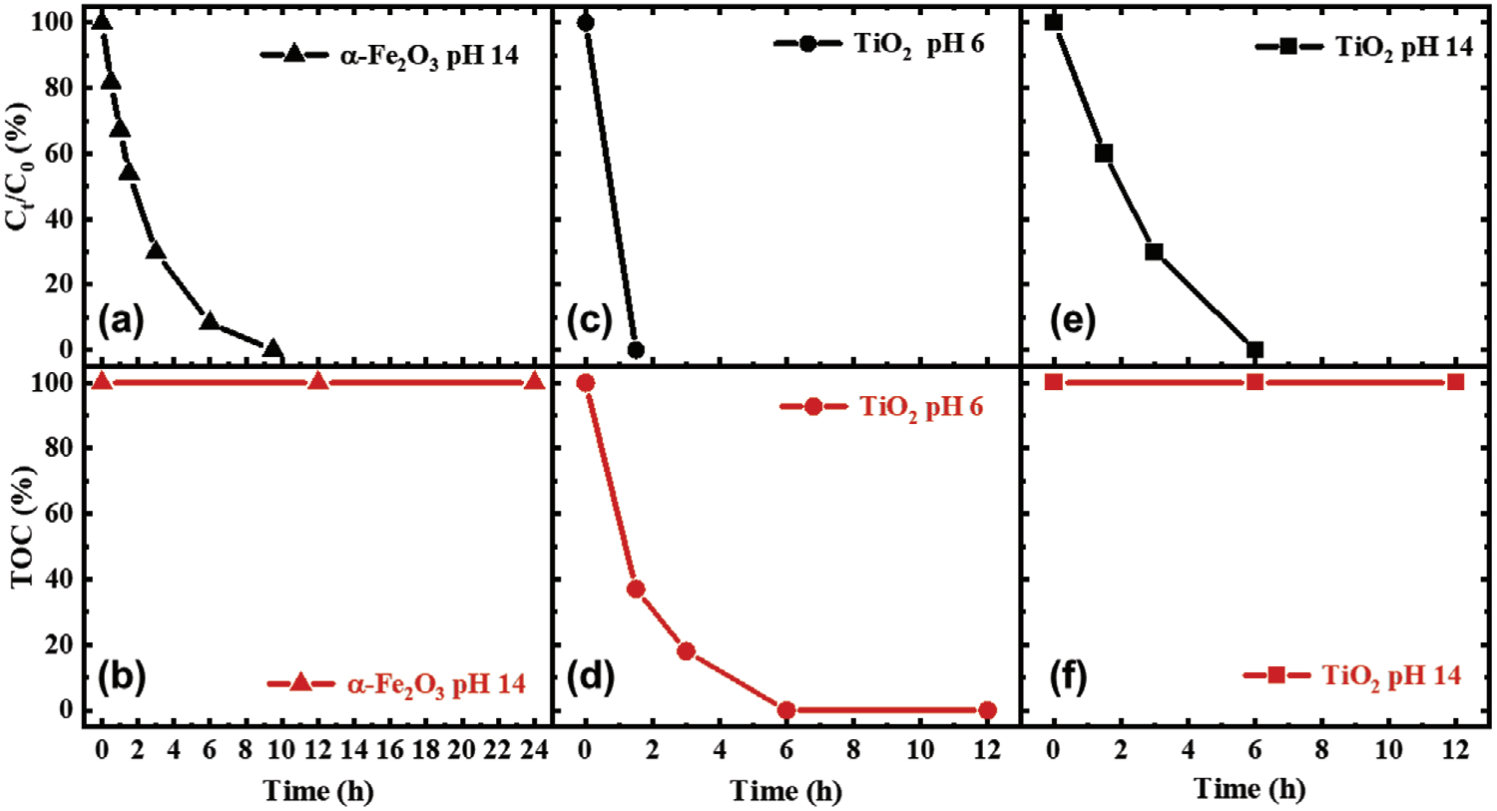
The percentage of MB degradation (upper) and TOC removal (lower) for the PEC degradation process on TiO_2_ (1.95 V_RHE_) and *α*‐Fe_2_O_3_ (1.62 V_RHE_) photoanodes under 365 nm illumination (intensity: 240 mW cm^−2^).

Despite higher photocurrent observed in both TiO_2_ and *α*‐Fe_2_O_3_, the oxidizability of MB on these two photoanodes still remains unclear. **Figure** **2**a,c directly compares the first oxidation (decoloration) of MB on TiO_2_ and *α*‐Fe_2_O_3_ measured under continuous 365 nm illumination. It is clear that both photoanodes can decolorate the MB electrolyte at low 5 ppm MB concentration measured by the UV–vis absorption as a function of time (Figure S4, Supporting Information), consistent with the PEC results with higher photocurrent. We note that *α*‐Fe_2_O_3_ exhibits a much slower decolorating rate due to smaller photocurrent. However, it is more important that the MB can be fully decolorated on *α*‐Fe_2_O_3_, indicating that it is capable of catalyzing MB degradation with the valence band potential at 2.5 V_RHE_.

**Scheme 1:**
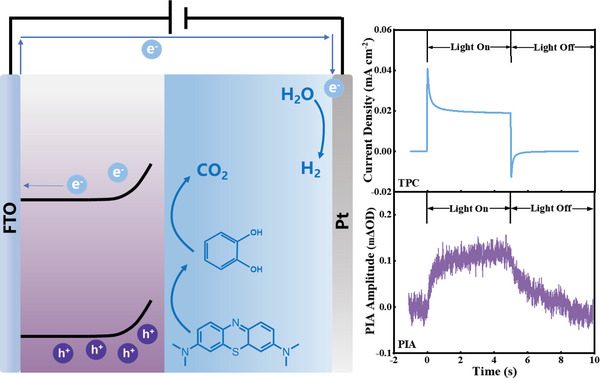
Schematic representation of the PEC oxidation of methylene blue and the corresponding typical PIA and TPC data measured during the reaction.

**Figure 3:**
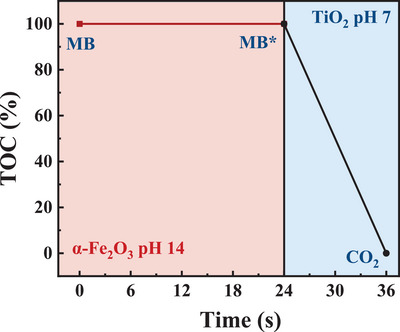
The percentage of TOC remaining in the electrolyte during PEC oxidation of MB. The PEC oxidation of MB (5 ppm, pH 14) was first carried out using the *α*‐Fe_2_O_3_ photoanode under 1.62 V_RHE_. The electrolyte was then neutralized to pH 7 for the TiO_2_ PEC oxidation under 1.95 V_RHE_. TOC was measured at t = 0, 24, and 36 h.

Although TiO_2_ and *α*‐Fe_2_O_3_ show its capability of MB decoloration, the further oxidation after decoloration is still unclear. Figure 2b,d shows the removal of TOC using both photoanodes as a function of time, indicating the overall mineralization of MB. For TiO_2_, the rate of MB mineralization (6 h) is much slower than that of decoloration (1.5 h), suggesting that the MB was firstly oxidized to small molecules (denoted as MB*) and secondly further oxidized to inorganic carbon. The O_2_ and H_2_ generated during bulk PEC MB and MB* oxidation were quantified as the faradaic efficiency to indicate whether photogenerated holes fully participated in the MB and MB* oxidation, without water oxidation as the side reaction. No oxygen was detected during the bulk PEC oxidation of MB and MB* in neutral pH (Figure S3a,d, Supporting Information) indicating that all photogenerated holes participated in the oxidation of MB and MB* without water oxidations. High‐performance liquid chromatography‐mass spectroscopy (HPLC‐MS) was carried out determining the structure of MB*. It shows that the MB was decomposed to catechol after decoloration (Figures S5 and S6, Supporting Information) illustrated in **Scheme** **1**. It is however striking to us that using *α*‐Fe_2_O_3_ in NaOH, the TOC in the MB electrolyte was not removed over 24 h. Instead, water oxidation appeared to be the dominant reaction with 100% O_2_ and H_2_ FE after MB decoloration shown in Figure S3e (Supporting Information), suggesting that MB* oxidation did not take place on the surface of *α*‐Fe_2_O_3_. HPLC‐MS indicated that the intermediate molecule measured after 24 h PEC oxidation of MB is identical to that oxidized by TiO_2_, suggesting that both MB oxidations for decoloration proceeded with the same reaction pathway. The difference in TOC removal is also supported by the PEC oxidation firstly using *α*‐Fe_2_O_3_ (and pH 14) for 24 h, then using TiO_2_ (pH neutralized to 7) to continue oxidizing the remaining electrolyte. It is clear, as shown in **Figure** **3**, that TiO_2_ can effectively remove all organic carbon from the MB intermediate originally generated by *α*‐Fe_2_O_3_. Such method of MB* synthesis was also used to prepare the electrolyte of MB* for the PEC oxidation of MB* using TiO_2_ as shown below.

We now turn to consider the pH impact upon the TOC removal on TiO_2_. Figure 2c,e compares the decoloration rate as a function of time using TiO_2_. It is clear that TiO_2_ can decolorate the MB electrolyte completely with 100% FE shown in Figure S3a and S3c (Supporting Information). However, the further oxidation of TOC removal cannot be proceeded in pH 14 electrolyte over a 12‐h PEC oxidation measurement (Figure 2c), in contrast to the TOC measured in pH 6 electrolyte with complete TOC removal after 6 h of PEC oxidation (Figure 2e). The FE measurements of MB and MB* oxidation by TiO_2_ in different pH also shows that mineralization of MB* was unable to be proceeded in pH 14, where oxygen evolution dominated the reaction with 100% FE opposite to the 0% FE in neutral pH for MB* oxidation. Such difference suggests that there is an underlying reaction competition between water oxidation and MB mineralization on TiO_2_ that determines the reaction pathway and oxidation selectivity, requiring detailed kinetic analyses and will be discussed below.

We now consider the oxidation selectivity on TiO_2_ and *α*‐Fe_2_O_3_. Photoinduced absorption (PIA) spectroscopy and transient photocurrent (TPC) were employed to determine the reaction kinetics of TiO_2_ and *α*‐Fe_2_O_3_ MB oxidation. Scheme 1 shows the representative PIA and the corresponding TPC responses monitored during PEC oxidations. The probe wavelengths of 475 and 650 nm were used to monitor the optical absorption change in TiO_2_ and *α*‐Fe_2_O_3_ photoanodes, respectively, following excitation on/off by 365 nm LED at each 5 s, which have been determined to be the photogenerated holes.^[^ ^4^, ^19^ ^]^ Previous transient and photoinduced absorption studies have indicated that the spectral feature of photogenerated holes was independent of the excitation intensities, suggesting that the photogenerated holes primarily accumulated in the valence band where the quasi Fermi level was approaching to the valence band edge.^[^ ^4^, ^19^ ^]^ Therefore, the thermodynamic driving force for these holes to oxidize water or MB/MB* molecules is invariant of the excitation intensity employed in this PIA study herein. The PIA data were calculated to the surface hole density of both TiO_2_ and *α*‐Fe_2_O_3_ using extinction coefficient of 2200 M^−1^ cm^−1^ and 672 M^−1^ cm^−1^, respectively (see Figure S7, Supporting Information for details and calculation). According to the surface hole density calculated from the PIA data, we note that the surface hole density was ≈0.1‐1 h^+^ nm^−2^, which is one order of magnitude higher than the surface defect state density reported for metal oxides.^[^ ^20^ ^]^ In addition, our previous study also revealed that the PIA spectra are independent of the excitation intensities, attributed to the surface accumulated holes in the space charge layer.^[^ ^4b^ ^]^ Therefore, the PIA signals observed in this work are primarily assigned to the surface hole density rather than the surface defect state for the PEC MB oxidation. Combining these PIA and TPC as a function of excitation intensities allows us to employ a simple rate law analysis to calculate the reaction order and rate constants, with the assumption of 100% FE for each reaction,^[^ ^15^, ^21^ ^]^ shown in Equation 1.

(1) $$\log\;J\;=\;\log\;k+\alpha \log[h^{+}]$$

where *J* is the oxidation reaction rate measured by the steady‐state photocurrent density (mA cm^−2^ or e^−^ s^−1^ nm^−2^), k is the reaction rate constant, *α* is the reaction order, and [*h* ^+^] is the density of surface holes (nm^−2^) accumulated at the photoanode surface.

**Figure 4:**
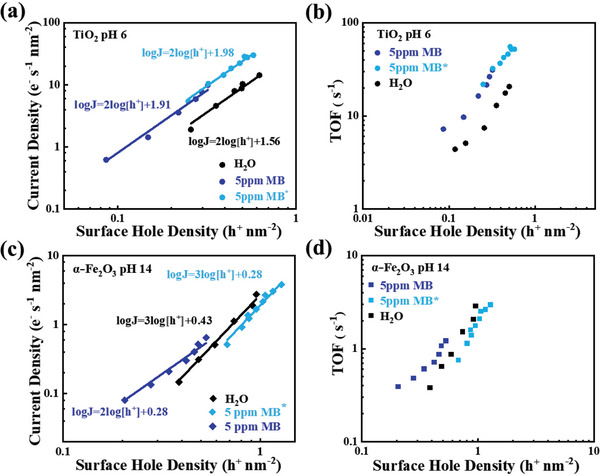
a) Rate law analysis of water oxidation (black), MB oxidation (blue) and MB* oxidation (cyan) at pH 6 at 1.95 V_RHE_ using the TiO_2_ photoanode. b) the comparison of TOF for MB (blue) and water oxidation (black) using the TiO_2_ photoanode. c) Rate law analysis of water oxidation (black), MB oxidation (blue) and MB* oxidation (cyan) at pH 14 at 1.62 V_RHE_ using the *α*‐Fe_2_O_3_ photoanode. d) the comparison of TOF for MB (blue), MB* (cyan) and water oxidation (black) using the *α*‐Fe_2_O_3_ photoanode.

**Figure** **4**a shows the rate law analysis of MB oxidation using TiO_2_ photoanodes. On the log‐log scale, all PEC oxidations (i.e., H_2_O, MB, MB*) show a linear relationship with photocurrent. It is clear that TiO_2_ shows a second‐order water oxidation kinetics in pH 6 electrolyte, in good agreement with a previous PIA study by Kafizas et al.^[^ ^19^ ^]^ Both MB and MB* oxidations also show the second order with higher reaction rate constant than water oxidation (also shown in Figure 4b and Figure S8, Supporting Information), consistent with the PEC results of higher photocurrent in the presence of organic molecules in Figure 1. It should be noted that both MB and MB* oxidations exhibit almost identical reaction kinetics. Figure 2 has shown that MB oxidation (decoloration) is different from MB* oxidation (mineralization). Therefore, despite the similar kinetics of MB and MB* oxidations on TiO_2_ photoanodes, the reaction pathway is different between these two oxidations according to the PEC results of mineralization and decoloration shown in Figure 2, as we discuss further below.

Figure 4c shows the rate law analyses of water, MB and MB* oxidations on the *α*‐Fe_2_O_3_ photoanode. A third‐order water oxidation reaction pathway in pH 14 NaOH was observed, consistent with other's reports and our previous study.^[^ ^4^, ^15^, ^21^, ^22^ ^]^ The reaction kinetics of MB decoloration, as also shown in Figure 4c, changes to a second‐order reaction, which is different from the third‐order water oxidation kinetics. In addition, despite the difference in reaction order, the effective turnover frequency (TOF) was calculated in order to directly compare these two reactions according to Equation 2.

(2) $$\mathrm{TOF}\;=\frac{\mathrm{current}\;\mathrm{density}}{\mathrm{surface}\;\mathrm{hole}\;\mathrm{density}}\;$$

where the current density (e^−1^ s^−1^ nm^−2^) was calculated from the steady‐state TPC data, and the surface hole density (h^+^ nm^−2^) was calculated from the steady‐state PIA amplitude at 5s.

It is apparent that the TOF for MB oxidation on TiO_2_ and *α*‐Fe_2_O_3_ is higher than the TOF for water oxidation. However, the MB* oxidation on TiO_2_ is significant faster than water oxidation, confirming the further oxidation of MB* to CO_2_. In contrast, *α*‐Fe_2_O_3_ can oxidize MB only to MB* according to the TOC measurement shown in Figure 2a,b. However, in the presence of MB*, the reaction on *α*‐Fe_2_O_3_ shows an identical third order to water oxidation shown in Figure 4c. Since the PEC results have indicated that the MB* oxidation was unable to take place in the pH 14 electrolyte, shown as the 100% TOC remaining over the 24‐h measurement in Figure 2b and 100% O_2_ evolution FE in Figure S3e (Supporting Information). It is therefore indicative that water oxidation is still the dominant reaction during the PEC MB oxidation using *α*‐Fe_2_O_3_ photoanodes after the MB molecules are oxidized to small organic catechol molecules (as MB*), clearly demonstrating the reaction and selectivity challenges for mineralization of MB using photocatalyst.

**Figure 5:**
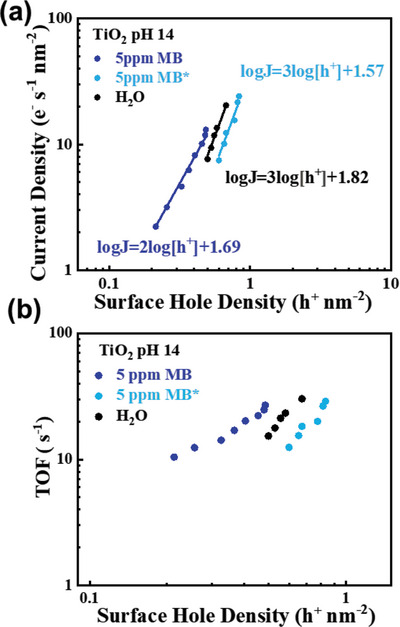
a) Rate law analysis of water oxidation (black), MB oxidation (blue) and MB* oxidation (cyan) at pH 14 at 1.62 V_RHE_ using the TiO_2_ photoanode. b) the comparison of TOF for MB (blue) and water oxidation (black) using the TiO_2_ photoanode.

**Scheme 2:**
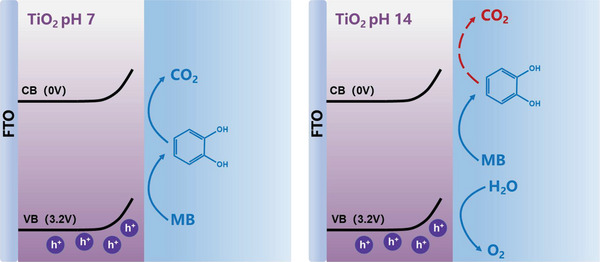
Schematic representation of the kinetic competition of water oxidation with MB and MB* oxidations in different pH on the surface of TiO_2_. Water oxidation is dominant reaction in pH 14, blocking the further oxidation of MB* to CO_2_.

Despite the mineralization of MB using TiO_2_, there is still a kinetic challenge using TiO_2_ for MB oxidation to CO_2_. **Figure** **5** shows the rate law analyses of TiO_2_ for water oxidation, MB oxidation and MB* oxidation. The water oxidation by TiO_2_ shows a third order reaction, consistent with the previous study.^[^ ^15^ ^]^ MB oxidation also exhibits a second order, in agreement with the MB oxidation measured in pH 6 shown in Figure 4a. However, in the presence of MB* at pH 14, the reaction order changed to third with similar rate constant of water oxidation, suggesting that water oxidation at pH 14 is the dominant reaction rather than MB* oxidation by TiO_2_ as illustrated in **Scheme** **2**, in perfect agreement with the 100% TOC remaining in the electrolyte despite the decoloration shown in Figure 2f. Based upon the PEC and kinetic results shown above, it is therefore clear that, although MB has been thought to be an easy and instable molecule (Figure S9, Supporting Information) to oxidize, the mineralization of MB certainly requires specific thermodynamic and kinetic conditions, which will be discussed below.

## 3 Discussion

### 3.1 Active Species for MB Oxidation on TiO_2_ and *α*‐Fe_2_O_3_ Photoanodes

Previous studies have assigned the PIA absorption to photogenerated holes at 460 and 650 nm for TiO_2_ and *α*‐Fe_2_O_3_ photoanodes for water and alcohol oxidations.^[^ ^4a^ ^]^ We first of all consider the photogenerated active species responsible for MB decoloration and mineralization.

Studies in literature have suggested that the main process of MB oxidation has been assigned to (1) direct oxidation by photogenerated holes, (2) by hydroxyl radicals from the first oxidation of H_2_O molecules, (3) singlet oxygen, or (4) by active oxygen by the reduction of oxygen molecules generated from water oxidation similar to a Fenton pathway.^[^ ^23^ ^]^ In our study reported herein, both TiO_2_ and *α*‐Fe_2_O_3_ photoanodes have been reported being able to oxidize water molecules to hydroxyl radicals by the photogenerated holes,^[^ ^15^, ^19^, ^21^, ^24^ ^]^ showing as the first‐order oxidation pathway. In the PEC measurement in Figure 1, the photocurrent was clearly increased in the presence of MB, strongly suggesting a rather different reaction pathway than water oxidation, otherwise the photocurrent should remain unchanged due to the unchanged reaction pathway of water oxidation. Therefore, it is highly unlikely that the MB oxidation proceeded from the intermediate oxidant, such as hydroxyl radical or active oxygen. In addition, such oxidation potential of the intermediate oxidants generated from different photoanodes, if there is any, should be invariant (e.g., 2.8 V_RHE_ for hydroxyl radicals^[^ ^25^ ^]^) regardless the difference of the valence band potential for photogenerated holes in TiO_2_ and *α*‐Fe_2_O_3_, which is in contrast to the variation of MB mineralization results using these two photoanodes. Therefore, the photogenerated holes are responsible for direct MB oxidation shown in PEC and PIA studies herein. However, the oxidizability of the photogenerated holes in TiO_2_ and *α*‐Fe_2_O_3_ varies with their respective valence band potential (i.e., oxidation power), that alters the oxidation pathway as we discuss in the following section.

### 3.2 Thermodynamic and Kinetic Control of the Reaction Pathway during PEC MB Oxidation

We now consider the reaction thermodynamics and kinetics that control the reaction pathway. Figure 2 shows that both TiO_2_ and *α*‐Fe_2_O_3_ are able to oxidize MB to a small organic molecule even at very low concentration of MB. This oxidation is attributed to breaking the chromophore monitored by UV–vis absorption and characterized by HPLC‐MS shown in supporting information Figures S5 and S6 (Supporting Information). It is apparent that both electrodes are efficient for this decoloration reaction by the photogenerated holes, evidenced by the faster kinetics than water oxidation shown in Figures 4, 5. These results are in good agreement with the literature reporting efficient photocatalysis of MB with the valence band potential greater than 1.6 V_RHE_.^[^ ^26^ ^]^

**Scheme 3:**
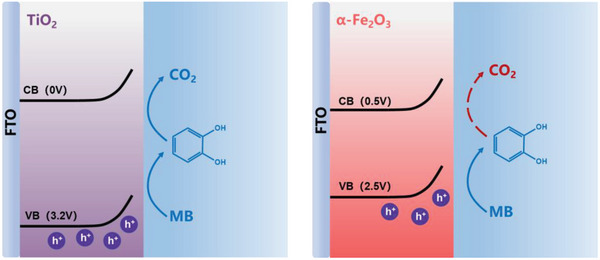
Schematic representation of the MB oxidation pathways for a) TiO_2_ and b) *α*‐Fe_2_O_3_ photoanodes used in this work.

The further oxidation, often associated with the continuous oxidation of the small molecules from MB and complete oxidation to CO_2_, has however attracted little attention in terms of the reaction mechanism. The observation, that the reaction kinetics change following the oxidation of MB* in TiO_2_, suggests that mineralization of MB on the surface of TiO_2_ takes place whilst the same reaction is unable to occur on the surface of *α*‐Fe_2_O_3_. Such observation is also supported by the TOC monitored during bulk PEC oxidation shown in Figure 2. Since the intermediate oxidants were ruled out as we discussed above, it is highly likely that the photogenerated holes in each material have different oxidizability to the MB* intermediate as summarized in **Scheme** **3**. Such difference in oxidizability is also likely associated with the valence band potential of TiO_2_ (3.2 V_RHE_) and *α*‐Fe_2_O_3_ (2.5 V_RHE_). The photogenerated holes in TiO_2_ are 700 mV deeper than that in *α*‐Fe_2_O_3_, providing significantly stronger oxidation power and thermodynamic driving force. The oxidation of MB* using TiO_2_ therefore remains higher reaction kinetics than water oxidation as shown in Figures 2d and 4a. In addition, such extra 700 mV oxidation power in TiO_2_ possibly enables the photogenerated holes to overcome the thermodynamic barrier as in *α*‐Fe_2_O_3_ to further oxidize MB*. In *α*‐Fe_2_O_3_, the water oxidation are kinetically challenging compared to TiO_2_.^[^ ^15^ ^]^ The MB* oxidation is kinetically more favorable for TiO_2_ in pH 6 due to the slower water oxidation. However, the slower kinetics of water oxidation on *α*‐Fe_2_O_3_, even in pH 14, should give rise to the oxidation of catechol. In contrast, the 100% O_2_ FE shown in Figure S3e (Supporting Information) clearly indicates that water oxidation is the primary reaction pathway regardless the slow reaction kinetics of water oxidation. Therefore, the inability of catechol oxidation by *α*‐Fe_2_O_3_ is likely to associate with the lack of thermodynamic driving force by the shallow valence band potential. We are also aware of the challenge in direct comparing the activation energies between MB and MB* oxidations due to the inactivity of MB* oxidation on α‐Fe_2_O_3_. However, we are able to qualitatively determine that TiO_2_ is more catalytic than *α*‐Fe_2_O_3_ according to the faradaic efficiencies and kinetic analyses, implying smaller activation energy required on TiO_2_. This observation is also consistent with our previous investigation of PEC alcohol oxidation using *α*‐Fe_2_O_3_ photoanodes, where aldehyde molecules oxidized from alcohol on *α*‐Fe_2_O_3_ is unable to be further oxidized due to significantly higher activation barrier (400 meV).^[^ ^4b^ ^]^ It is therefore clear that the oxidation of MB* also requires higher thermodynamic driving force, either by overcoming the activation barrier or by deeper valence band potential such as TiO_2_ that is intrinsically more in favor for mineralization.

Previous kinetic studies have indicated that water oxidation on TiO_2_ photoanodes is kinetically faster than that on *α*‐Fe_2_O_3_ due to the deeper valence band potential at 3.2 V_RHE_. Such valence band potential not only provides higher thermodynamic driving force for water oxidation, but also efficiently suppresses the surface recombination, that severely limits the water oxidation efficiency on BiVO_4_ and *α*‐Fe_2_O_3_ with valence band potential ≈2.5 V_RHE_.^[^ ^15^, ^19^, ^27^ ^]^ However, in our study herein, it is striking to us that the amplitude of valence band potential completely alters the reaction pathway of organic oxidations, strongly suggesting that the thermodynamic challenge is the key consideration for the functionality of photocatalysts, especially for the oxidation of organic molecules.

Apart from the semiconductor's valence band potential that provides the thermodynamic driving force for the photogenerated holes, the reaction kinetics are considered for the reaction pathway of PEC MB oxidation. Our PEC results have indicated that TiO_2_ is able to fully oxidize MB molecules to inorganic carbon in pH 6 electrolyte. Rate law analyses based upon the PIA results also show that the MB oxidation is kinetically faster than water oxidation despite the low concentration of MB employed in this study. The reaction kinetics shown in Figure 4a, however, exhibit a clear enhancement of reaction rate constant compared to water oxidation and Figure S3 (Supporting Information) shows all photogenerated holes were able to conditionally participating in the oxidation of MB and MB*. It is thus concluded that the concentration of MB (5 ppm) employed in this study is sufficient to distinguish the kinetic difference between MB oxidation and water oxidation in both PEC and PIA measurements.

The comparison of the MB mineralization using TiO_2_ photoanodes between pH 6 and 14 suggests that there is a kinetic difference for the photogenerated holes to oxidize MB molecules. It is particularly important to note that the photogenerated holes are unable to oxidize the MB molecule to CO_2_ in the pH 14 electrolyte. The third‐order reaction of water oxidation on TiO_2_ in pH 14 shown in Figure 5a is a clear indication of the reaction pathway being different from the MB to MB* oxidation (i.e., the second order). The third order reaction observed in the presence of MB* suggests that water oxidation is the dominant reaction pathway and the further oxidation of MB* does not occur, in contrast to the second‐order reaction of MB* oxidation measured in pH 6 in Figure 4a. It is therefore clear that MB* oxidation is unable to kinetically compete with water oxidation in pH 14, leading to the incomplete mineralization of MB on TiO_2_ photoanodes, regardless the sufficient thermodynamic driving force from the valence band potential in TiO_2_.

Our observation of the kinetic competition of MB* oxidation versus water oxidation on TiO_2_ photoanodes suggests that the kinetics of water oxidation differs from electrochemical environment. Water oxidation on metal oxides often requires proton‐coupled electron transfer (PCET) to facilitate charge transfer and energetically regulate the surface, for example redox levelling.^[^ ^15^, ^22^ ^]^ This PCET process is promoted at higher pH due to the more favorable reaction to extract protons and to reach the rate‐determining step of water oxidation, which has been observed and characterized on the surface of *α*‐Fe_2_O_3_^[^ ^15^, ^28^ ^]^ and TiO_2_.^[^ ^19^ ^]^ Our observation of water oxidation kinetics on TiO_2_ photoanodes is also in good agreement with these previous studies in terms of turnover frequency and reaction order, indicating that such conclusion of water oxidation is independent of the method of photoanode fabrication and the semiconductor properties (e.g., donor densities, trap states etc).

On the other hand, the oxidation of MB* to CO_2_ is significantly affected by the kinetics of water oxidation. It has been observed above, that the first oxidation of MB to MB* is a kinetically faster reaction than water oxidation, which has been also observed with decoloration independent of pH, also consistent with the literature employing MB as the indicator for photocatalysis. ^[^ ^29^ ^]^ However, our PIA and rate law analyses clearly indicate that there is a kinetic challenge of MB* oxidation in which the reaction pathway is determined by the kinetic competition of water oxidation. The oxidation of MB* is not a kinetically fast reaction to competing with water oxidation, based upon the difference in TOC measured in pH 6 and 14. The point of zero charge of TiO_2_ is c.a. 6–7. Previous studies have suggested that the 2^nd^ order water oxidation on TiO_2_ in the pH neutral electrolyte is associated with the 2^nd^ hole reacting with the surface hydroxyl radicals.^[^ ^19^ ^]^ In our work, since the TiO_2_ surface is dominated by the water molecules in pH 6, the dissociation and deprotonation of water molecule becomes a kinetically challenging reaction, thus giving rise to the oxidation of catechol proceeding as the mineralization. On the other hand, in pH 14, the surface is fully covered by OH^−^, the water oxidation is therefore proceeded with three neighboring holes, even in the presence of catechol (MB*), shown in Figure 5. At pH 6, water oxidation is limited by the deprotonation evidenced by the second‐order reaction and slower reaction kinetics,^[^ ^19^ ^]^ the MB* is therefore to be oxidized by the photogenerated holes. However, at pH 14, the water oxidation shifts to a faster reaction pathway, resulting in the MB* oxidation being no longer kinetically competitive. Photogenerated holes therefore are more in favor of oxidizing water molecules rather than MB* despite more favorable thermodynamic driving force. Our observation of this kinetic control by the reaction kinetics is also in agreement with the previous study of reaction competition of ethanol and acetaldehyde oxidation on hematite photoanodes.^[^ ^4a,b^ ^]^ The reaction pathway is controlled by the kinetics of the two competing reactions, as the result of different activation energies.^[^ ^4b^ ^]^ In our study herein, it is then clear that the MB* oxidation is more catalytic than the second‐order water oxidation in pH 6, however less than the third‐order water oxidation in pH 14, also indicating that the importance of the kinetic consideration for effective and efficient organic oxidations.

Our kinetic study herein demonstrates that although the photogenerated holes in metal oxide semiconductors are highly oxidizing due to the deep valence band potential, the oxidation of organic molecules is still of great challenge due to unexpected kinetic competition. The MB employed in this study as the model reaction has been thought to be relatively easy to be oxidized and thus frequently used as the evidence for photocatalytic dye degradation. This easy compound is even instable over prolonged storage in the air as the UV–vis absorption changes shown in Figure S9 (Supporting Information), suggesting a degradation of the MB molecules likely due to oxidation by oxygen in the air. However, the further oxidation of these molecules, especially aiming to fully oxidize to CO_2_, has demonstrated the great difficulty due to either lack of thermodynamic driving force (by *α*‐Fe_2_O_3_) or sluggish oxidation kinetics (by TiO_2_ in pH 14). Previous study of methanol oxidation has suggested that H‐C activation in the CH_3_OH molecule is essential for the formation of ‐C = O bond as the rate determining step.^[^ ^4a^ ^]^ However, in our study reported herein, the alcohol carbon center in the catechol are fully occupied with C‐C and C = C bond. Therefore, the oxidation of catechol molecule would directly involve with the breakage of C‐C or C = C bond first in order for the ‐OH group to be oxidized, which apparently requires higher energy and faster reaction to overcome both thermodynamic and kinetic barriers when competing with water oxidation. This conclusion is also extended to a more general concern of organic oxidation, especially to the breakage of C‐C bond in organic molecules, using semiconductors for photoinduced oxidations. Our previous PIA study of ethanol oxidation on *α*‐Fe_2_O_3_ photoanodes has shown that the acetaldehyde is the final product from ethanol with a second‐order reaction mechanism,^[^ ^4b^ ^]^ which is consistent with the methanol oxidation to formaldehyde,^[^ ^4a^ ^]^ indicating that the oxidation by the photogenerated holes in *α*‐Fe_2_O_3_ does not involve with C‐C bond breaking. These previous studies further support our conclusion of this study that the oxidation of C‐C bond must consider both the thermodynamic and kinetic advantages to provide sufficient driving force, and to accelerate reaction to minimize the side reaction pathway and the resulted by‐product formation.

Our optical spectroscopic study of TiO_2_ and *α*‐Fe_2_O_3_ for MB oxidation strongly suggest the consideration of thermodynamic and kinetic competition for the mineralization reaction, rather than simply decolorating the MB electrolyte. The limitation for the deep oxidation of organic molecules has been reported with incomplete TOC removal for many organic substrates including organic dyes^[^ ^30^ ^]^ and anti‐biotics,^[^ ^10^, ^11^, ^31^ ^]^ using the photocatalysts with modest valence band potential ≈1.6‐2.5 V_RHE_. Such oxidation power, however, is insufficient to oxidize the small organic intermediate molecules to CO_2_. On the other hand, Li et al reported a nanocone structure of TiO_2_ to oxidize persistent pollutants of 4‐cholorophenol with 100% TOC removal.^[^ ^7a^ ^]^ In addition, they also showed that WO_3_ is able to mineralize bisphenol A (BPA) in a PEC configuration.^[^ ^7b^ ^]^ Both valence band potentials of TiO_2_ and WO_3_ are > 3 V_RHE_, in perfect agreement with our proposed thermodynamic driving force required for organic oxidation.

In addition to the thermodynamic requirement, the kinetic competition is also often overlooked in organic oxidations. Water oxidation in the presence of organic oxidation is considered as a side reaction that consumes photogenerated holes thus limiting the efficiency. However, our results of MB oxidation using TiO_2_ clearly demonstrate that water oxidation is a dominant reaction pathway in pH 14 simply due to faster reaction kinetics than MB mineralization. On the other hand, the kinetics of organic oxidation on photocatalysts with shallow valence band potential (e.g., 2 V_RHE_) can be accelerated by modifying the photoanode surface with co‐catalysts. Stolarczyk et al. demonstrated the full oxidation of methanol to carbonate using Pt as the co‐catalyst on g‐C_3_N_4_.^[^ ^5^ ^]^ Since g‐C_3_N_4_ is thermodynamically unfavorable for complete oxidation of alcohol,^[^ ^18^ ^]^ the primary function of Pt is to change the reaction pathway of methanol oxidation with 100% efficiency. Semiconducting photocatalysts with visible light absorption have great absorption range, giving rise to the maximum efficiency of organic oxidation, the valence band potential is however often ≈1.5‐2.5 V_RHE_, that is difficult to fully oxidize organic molecules. The function of co‐catalysts, therefore, is promising for the broad spectrum of light absorption and meanwhile the mineralization of organics with maximum efficiency.

This study addressed the challenges for alternative oxidation using metal oxide photoanodes. We used methylene blue as the model molecule that has been considered a simple reaction. However, the PIA studies reveal that the complete oxidation to CO_2_ is not as simple as it decolorates, clearly showing the underlying limitation and challenges in the inherent properties of photocatalysts in their thermodynamics and kinetics. This model concluded from the spectroscopic results can also be extended to other PEC or photocatalytic organic oxidation systems such as methanol and biomass reforming for hydrogen production, ^[^ ^32^ ^]^ environmental catalysis of pollutants or persistent compounds^[^ ^7^ ^]^ from chemical industry, all of which require to completely break down the organic molecules to CO_2_.

## 4 Conclusion

In this paper, we have focused on the reaction pathway of MB oxidation on TiO_2_ and *α*‐Fe_2_O_3_ photoanodes employing photoinduced absorption spectroscopy. The decoloration of MB to small molecules is proceeded with direct oxidation by the photogenerated holes in both TiO_2_ and *α*‐Fe_2_O_3_. The further mineralization of these small molecules to CO_2_, on the other hand, exhibits different reaction pathways depending on oxidation conditions applied in the system. The mineralization on *α*‐Fe_2_O_3_ is unable to occur due to the modest valence band potential (2.5 V_RHE_) that is insufficient to provide thermodynamic driving force. TiO_2_ can mineralize MB in neutral pH electrolyte whilst water oxidation occurs after decoloaration in pH 14 electrolyte due to faster kinetics of photogenerated holes for water oxidation. Our results indicate that the oxidation of organic molecules is not a simple reaction using photocatalysis and photoelectrochemistry due to the thermodynamic and kinetic challenges. Proposed solutions to these challenges include employing deep valence band semiconductors to have greater driving force between the oxidation potential of organics and the photogenerated holes, or modifying the modest valence band semiconductor with efficient cocatalysts to completely change the reaction pathway and to have absolute kinetic advantage of the oxidation on the cocatalyst. Careful consideration should be given to the relative kinetics and the thermodynamic driving force by the semiconductor and photocatalyst.

## 5 Experimental Section

### Materials

Acetic acid (>99.5%) was purchased from Titan. Sodium hydroxide (NaOH, >96.0%), acetylacetone (99%), and sodium sulfate (Na_2_SO_4_, >99.0%) were purchased from Sinopharm Chemical Reagent. Iron(III) acetylacetonate (>98%) was purchased from Shaoyuan. Titanium butoxide (≥99%) was purchased from Macklin. Fluorine‐doped tin oxide (FTO, resistance < 15 Ω sq^−1^, transmittance >83%) substrates were purchased from Kaivo Optoelectronic Technology.

### Fabrication of the TiO_2_ Photoanodes

The method of fabrication of undoped TiO_2_ photoanodes was used with modifications from the metal‐organic deposition method previously reported for BiVO_4_ thin film fabrication.^[^ ^27^ ^]^ A sol‐gel precursor was prepared by dissolving titanium butoxide (3.91 mL) in acetic acid (1.5 mL) and acetylacetone (10 mL). A piece of FTO (3 cm × 2.5 cm) was cleaned by sonication with glass cleaner (Shanghai Johnson) in deionized water (18.25 MΩ cm at 25 °C, Ereeran, YL400BU). The FTO was then sonicated in 95% ethanol before calcined at 450 °C (Nabertherm, LE 6/11/R7) for 30 min to remove all organic residues.

TiO_2_ films were fabricated by depositing 100 µL of the precursor solution on the FTO by a spincoater (Laurel, WS‐650Mz‐23NPPB). Spincoating parameters were set at 1000 rpm for 30 s for each layer of deposition. For each layer, a small piece of the FTO substrate was covered with a piece of tape to leave blank FTO (0.5 cm ×3 cm) for electrical connection. After each layer was deposited, the film was calcined at 580 °C for 10 min and cooled at room temperature. This spincoating/calcination procedure was repeated 12 times. After depositing the 12th layer, the coated film was calcined at 580 °C for 3 h.

### Fabrication of *α*‐Fe_2_O_3_ Photoanodes

A sol‐gel precursor was obtained by dissolving iron(III) acetylacetonate (0.1 mol L^−1^) in acetic acid (1.5 mL) and acetylacetone (10 mL). The precursor solution was magnetically stirred at room temperature (25 °C) for 30 min. The size and preparation process of FTO are the same as above TiO_2_ photoanodes.

The *α*‐Fe_2_O_3_ film was fabricated by depositing 100 µL of the precursor solution on the FTO by a spincoater (Laurel, WS‐650Mz‐23NPPB). Spincoating parameters were set at 1000 rpm for 30 s for each layer of deposition. For each layer, a small piece of the FTO substrate was covered with a piece of tape to leave blank FTO (0.5 cm × 3 cm) for electrical connection. After each layer was deposited, the film was calcined at 580 °C for 10 min and cooled at room temperature. This spincoating/calcination procedure was repeated 9 times. After depositing the 9th layer, the coated film was calcined at 580 °C for 3 h.^[^ ^4b^ ^]^

### Characterizations

The XRD data were recorded by an X‐ray diffractometer (Haoyuan, DX‐2700BH) using Cu K*α* radiation (*λ* = 1.5406 Å). UV–vis spectra were recorded using UV–vis spectrophotometer (Shimadzu, UV‐1099i). The SEM images of the TiO_2_ and *α*‐Fe_2_O_3_ samples were recorded by a Hitachi field‐emission scanning electron microscope (S‐4800) with acceleration voltage of 5 kV. Total organic carbon (TOC) of the solution was measured via a total organic carbon analyzer (Analytik Jena, Multi N/C 3100). The product of PEC oxidation of MB was separated and determined using the high‐performance liquid chromatography‐mass spectroscopy (Series Triple Quadrupole 1290–6470B, Agilent). The column (ZORBAX Eclipse Plus C‐18 RRHD) temperature was 30 °C. The carrier was a methanol‐water mixture (90:10, v/v). The injection volume was 2 µL with the flow rate of 0.4 mL min^−1^.

### PEC Oxidation of Methylene Blue

The PEC oxidation of water and MB measurements were carried out in a custom‐made transparent quartz reactor. Autolab potentiostat (PGSTAT204) was used to record the electrochemical responses. One 365 nm LED light (LG, XP‐3535) was used as the light source with the intensity indicated where needed. The three‐electrode configuration was constructed using the TiO_2_ or *α*‐Fe_2_O_3_ as the working electrode, a platinum mesh (1.5 cm × 1.5 cm) as counter electrode and a Ag/AgCl as reference electrode. The electrolyte (50 mL) consisted of 5 ppm MB and 0.1 m Na_2_SO_4_ for PEC measurements using TiO_2_ and 1 m NaOH for *α*‐Fe_2_O_3_. Back‐side illumination was used in the PEC and PIA measurements, where the electrode was fixed on the wall of the customized quartz electrolytic cell. The TiO_2_ or *α*‐Fe_2_O_3_ was in contact with the electrolyte with a fixed area of 0.785 cm^2^. In PEC MB degradation of TOC measurements, the illuminated photoelectrode area was enlarged to 6.25 cm^2^ for both TiO_2_ and *α*‐Fe_2_O_3_ photoanodes with the front‐side illumination.

### Determination of the Faradaic Efficiency of MB and MB* Oxidations

The quantification of O_2_ and H_2_ evolution during PEC MB and MB* oxidation using TiO_2_ and *α*‐Fe_2_O_3_ photoanodes, and the calculation of corresponding faradaic efficiency were reported previously.^[^ ^4^, ^33^ ^]^ In brief, the PEC oxidation in the three‐electrode configuration was carried out using a gas‐tight gas product circulation and collection system (Labsolar 6A, Beijing Perfectlight). The gas product was generated in the system and sent automatically to a gas chromatography (GC, GC9790 II, FuLi Instruments). The system was initially vacuumed to 1 kPa for the O_2_ and H_2_ continuously generated to accumulate in the system. Due to the low pressure in the system, the temperature of the reactor was estimated to be 278 K. The sampling volume of the gas mixture for GC quantification was 0.6 mL compared to 600 mL of the total volume. The sampled H_2_ and O_2_ were sent to a RubyBond 5A column by argon carrier at 373 K, and detected by a thermal conductivity detector at 423 K. The calibration of H_2_ and O_2_ was carried out using 99.999% H_2_ from an alkaline electrolyzer (SPH‐300, Beijing BCHP) and 99.999% O_2_ (Air Liquide).

The faradaic efficiency was therefore calculated using the following equation:

(3) $$\mathrm{FE}\;=\frac{\mathrm{charges}\;\mathrm{for}\;\mathrm{gas}\;\mathrm{evolution}}{\mathrm{total}\;\mathrm{charges}\;\mathrm{generated}}\;=\frac{n\times z\times F}{I\times t}\;\times 100\%$$

where *n* (mol) is the moles of H_2_ or O_2_ generated, *I* (*A*) is the photocurrent during the measurement, *t* (*s*) is the reaction time, *z* is the number of electrons in the O_2_ (*z* = 4) and H_2_ (*z* = 2) evolution reactions, and *F* is the faradaic constant (96 485 C mol^−1^).

### Photoinduced Absorption Spectroscopy of PEC Water, MB and MB* Oxidations

The operando spectroelectrochemical photoinduced absorption spectroscopy was modified from the previous report.^[^ ^21a^ ^]^ The above‐mentioned quartz PEC cell was used for PIA measurement. The applied potential was controlled and transient photocurrent data recorded using a CHI potentiostat (1140C). The long pulse 365 nm LED was used to excite the TiO_2_ and *α*‐Fe_2_O_3_ photoanode. The 5 s on/off pulse was controlled by a frequency generator (Junce Instruments, JDS660). A 150 W tungsten filament lamp with a monochromator (Zolix Instruments, TLS15‐T150A) used as the probe light source. The monochromatic probe light was filtered through long‐pass filters (Hengyang Optics) and a band‐pass filter (Rayan Optics) at the specified probe wavelength and then recorded by a homemade silicon photodiode (Hamamatsu S5973). Optical data was sent to a data acquisition device (National Instruments, USB‐6361) for processing on the Labview platform.^[^ ^4b^ ^]^

## Conflict of Interest

The authors declare no conflict of interest.

## Supporting information

Supporting Information

## Data Availability

The data that support the findings of this study are available from the corresponding author upon reasonable request.
