# Supplementary material for: Photoinduced Absorption Spectroscopy of Photoelectrocatalytic Methylene Blue Oxidation on Titania and Hematite: The Thermodynamic and Kinetic Impacts on Reaction Pathways
Source: Adv Sci (Weinh). 2023 Jan 22;10(9):2206685. doi: 10.1002/advs.202206685 (PMC10037980; doi:10.1002/advs.202206685)
Supplement: Supplementary file 1 — Supporting Information [file ADVS-10-2206685-s001.pdf]

ADVANCED SCIENCE
Open Access

Supporting Information

for *Adv. Sci.*, DOI 10.1002/advs.202206685

Photoinduced Absorption Spectroscopy of Photoelectrocatalytic Methylene Blue Oxidation on Titania and Hematite: The Thermodynamic and Kinetic Impacts on Reaction Pathways

*Xinyue Guo, Zixuan Ma, Yuling Yuan, Yan Kang, Hong Xu, Zhiping Mao and Yimeng Ma**

Supporting Information

Photoinduced Absorption Spectroscopy of Photoelectrocatalytic Methylene Blue Oxidation on Titania and Hematite: The Thermodynamic and Kinetic Impacts on Reaction Pathways

Xinyue Guo[a], Zixuan Ma[a], Yuling Yuan[a], Yan Kang[c], Hong Xu[a], Zhiping Mao[a,b], and Yimeng Ma[a,b]*

[a]Key Laboratory of Science and Technology of Eco-Textile, Ministry of Education; College of Chemistry and Chemical Engineering, Donghua University, Shanghai, 201620, China.

[b]National Innovation Center of Advanced Dyeing & Finishing Technology, Shandong Zhongkang Guochuang Research Institute of Advanced Dyeing & Finishing Technology Co., Ltd., Taian City, Shandong Province, 271000, China.

[c]Shanghai Jahwa United Co., Ltd., Shanghai, 200082, China.

Corresponding Author:

*Correspondence: yimeng.ma@dhu.edu.cn

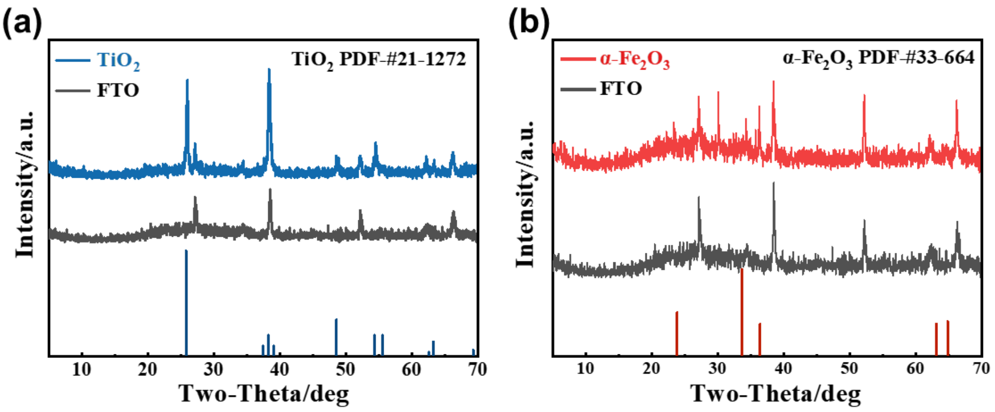

Figure S1. XRD data of (a) TiO_2 (blue) and (b) α-Fe_2O_3 (red) photoanodes.

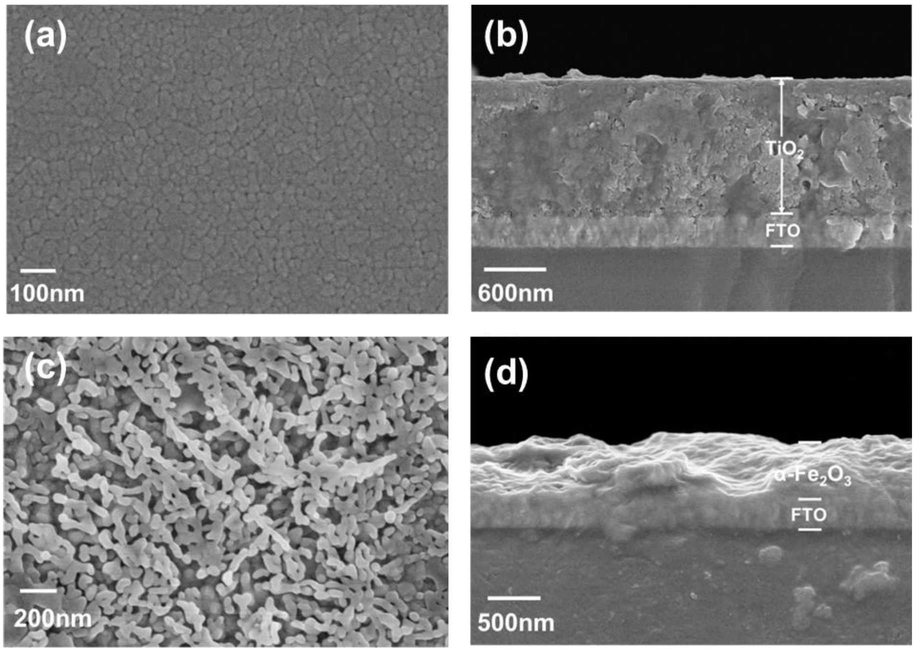

Figure S2. SEM images of photoanodes. (a) Top view of TiO_2, (b) Cross-sectional view of TiO_2, (c) Top view of α-Fe_2O_3, (d) Cross-sectional view of α-Fe_2O_3.

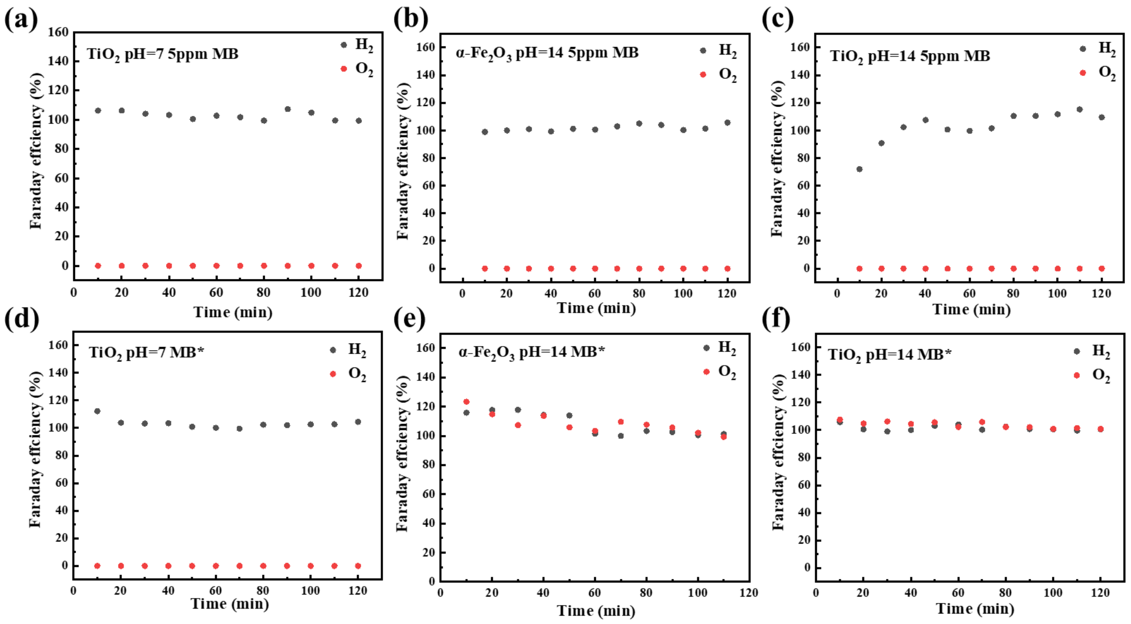

Figure S3. Faradaic efficiencies of H_2 and O_2 evolution in 5 ppm MB and MB* electrolytes. Upper: PEC oxidation of 5 ppm MB using (a) TiO_2 in pH 7, (b) α-Fe_2O_3 in pH 14 and (c) TiO_2 in pH 14; Lower: PEC oxidation of MB* using (d) TiO_2 in pH 7, (e) α-Fe_2O_3 in pH 14 and (c) TiO_2 in pH 14. Applied potential: 1.95 V_{RHE} for TiO_2 and 1.62 V_{RHE} for α-Fe_2O_3 photoanodes. Excitation intensity: 5.3 mW cm^{-2}.

The degradation of methylene blue by TiO_2 and α-Fe_2O_3 was characterized by UV-vis:

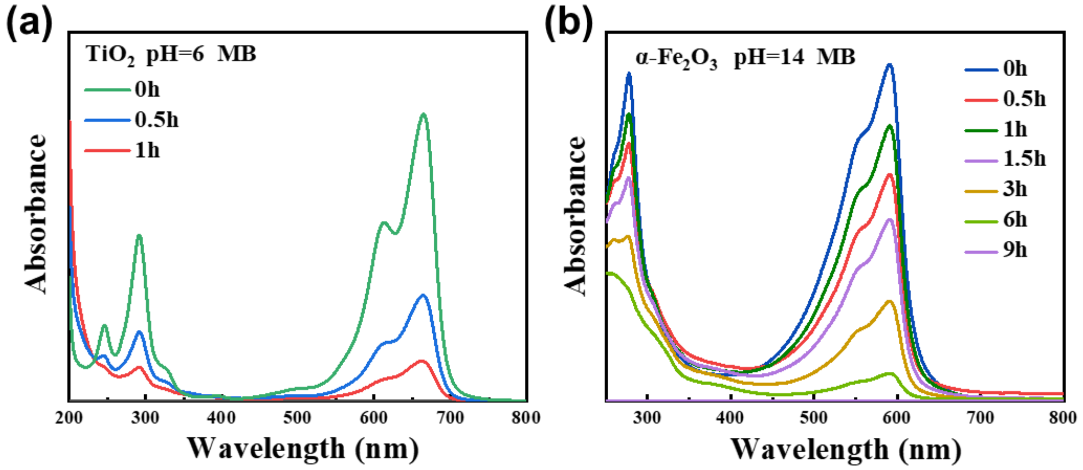

Figure S4. The UV-vis of MB degradation for the PEC degradation process on (a) TiO_2 (1.95V_{RHE}) and (b) α-Fe_2O_3 (1.62V_{RHE}) photoanodes.

HPLC-MS analyses:

The HPLC-MS measurement was carried out to characterize the structure of methylene blue and the subsequent oxidation products. Figure S5 shows the MS data of MB's m/z peak at 0.382

min of HPLC retention time. Both MB oxidation products by TiO_2 and α-Fe_2O_3 photoanodes shows remarkable identical small molecule fragments. We are able to identify the m/z 131 peak as the sodium chelated catechol at the HPLC retention time at 0.307 min for TiO_2 (Figure S6a) and 0.34 min for α-Fe_2O_3 (Figure S6b). These data suggest that the methylene blue was decomposed to be catechol as the MB* intermediate.

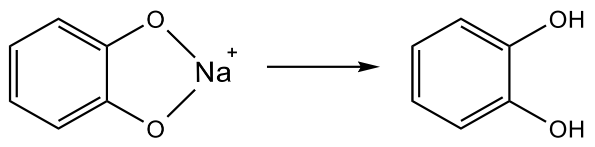

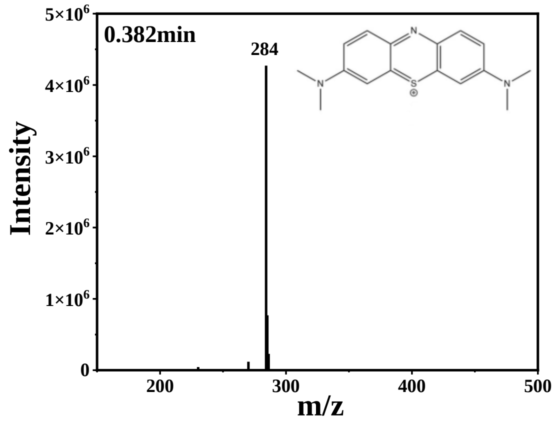

Figure S5. LC-MS spectrum of MB.

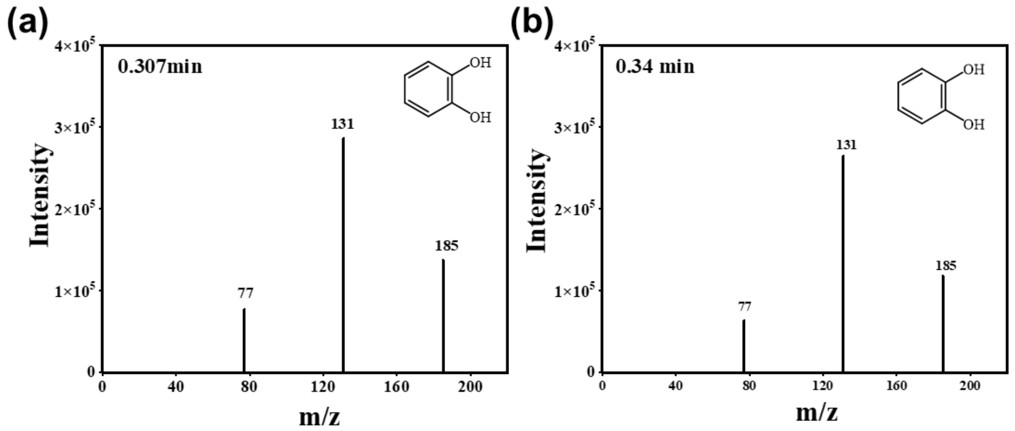

Figure S6. Mass spectra of major intermediates of MB dye degradation after 24h on (a) TiO_2 (1.95V_{RHE}) and (b) α-Fe_2O_3 (1.62V_{RHE}) catalysts. The m/z 131 peak detected is associated with a sodium chelated catechol cation in $NaSO_4$ and NaOH electrolytes detected by the MS.

Calculation of extinction coefficients of the photogenerated holes in TiO_2:

The extinction coefficient of TiO_2 photogenerated holes was determined using photoinduced

absorption spectroscopy. An potential was set at which back electron/hole recombination was dominating the PIA amplitude. The photocurrent response measured concurrently represents the amount of charges for back electron/hole recombination. Therefore, the extinction coefficient can be obtained using Beer-Lambert Law:

$$\Delta OD = \varepsilon_{h^+} \times P_S$$

where ΔOD is the PIA absorbance of the surface photogenerated holes; ε_{h^+}(M^{-1} cm^{-1}) is the extinction coefficient of the photogenerated holes; P_S (nm^{-2}) is the hole density on photoelectrode surface.

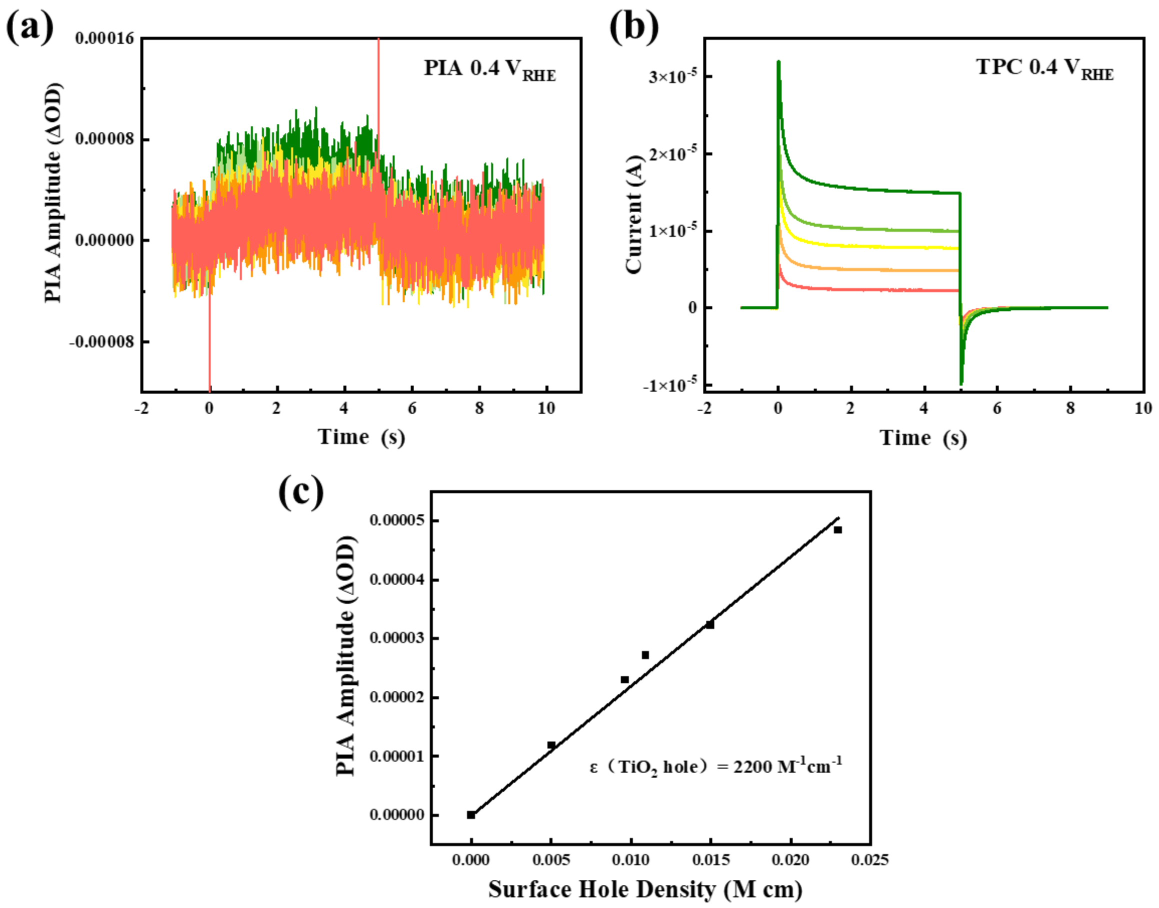

Figure S7. Extinction coefficient of TiO_2 in 0.1 M Na_2SO_4 at pH=6. (a) PIA amplitude of TiO_2 during electron/hole recombination and (b) the corresponding TPC response measured at 0.4 V_{RHE} at various 365nm light intensities; (c) the relationship of surface hole density and PIA amplitude, with the slope indicating the extinction coefficient of TiO_2 photogenerated holes is 2200 $M^{-1}cm^{-1}$.

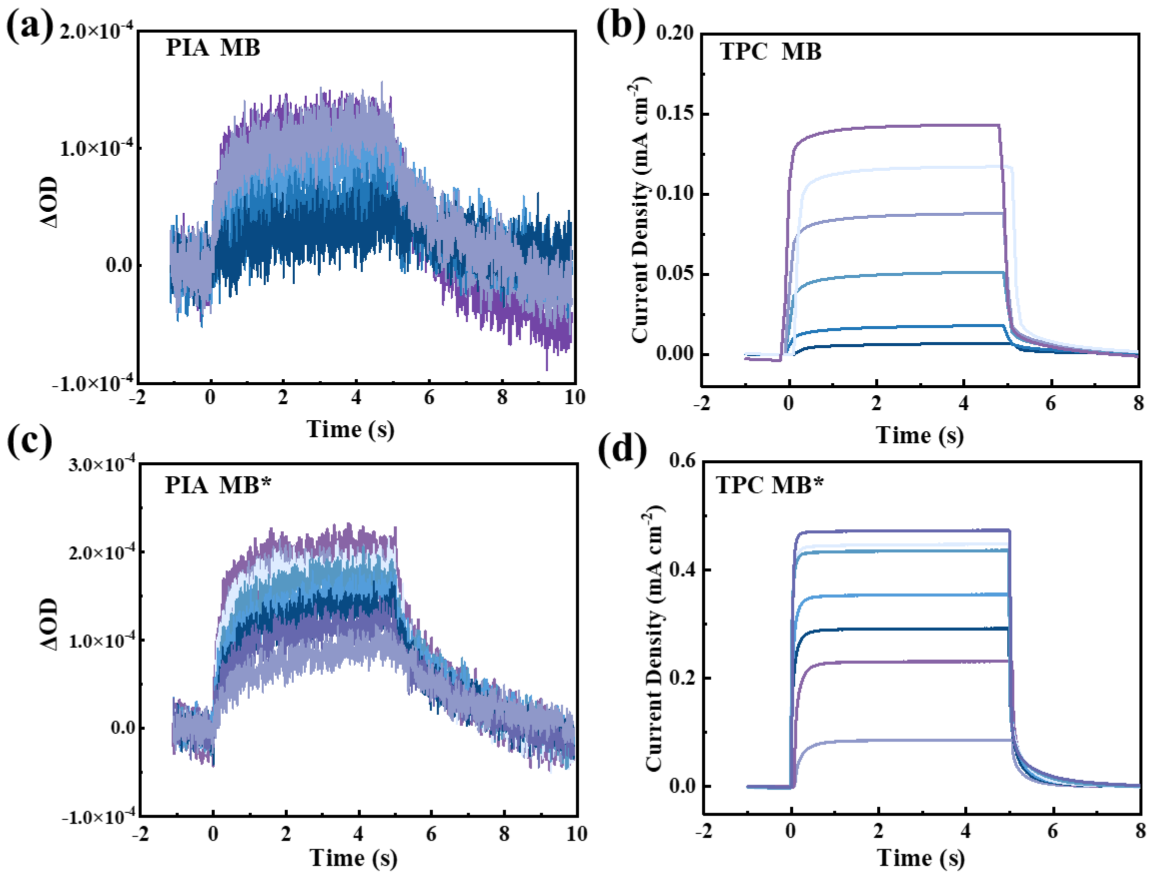

Figure S8. PIA and TPC data of TiO_2 oxidation in (a) MB and (b) MB* in 0.1 M Na_2SO_4 aqueous solution (pH 6) at 1.95 V_{RHE} during 5 s on/off pulse under 365 nm illumination conditions.

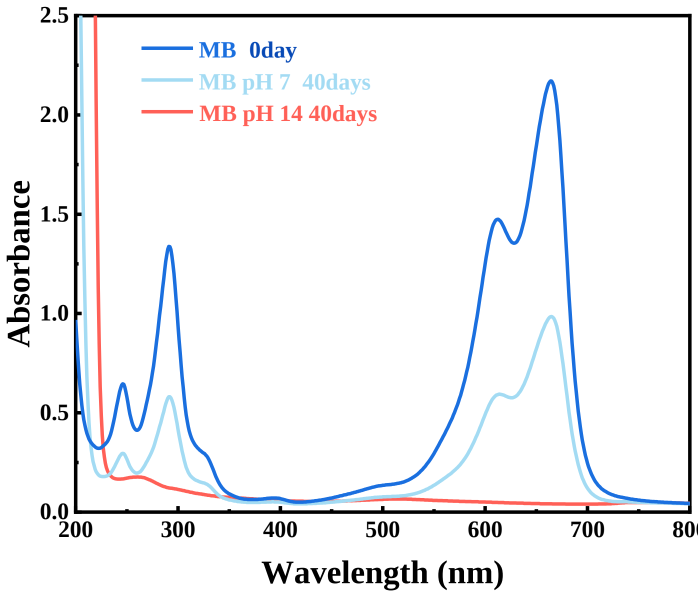

Figure S9. The UV-vis of newly configured 5ppm methylene blue and 5ppm methylene blue after 40 days of natural standing.

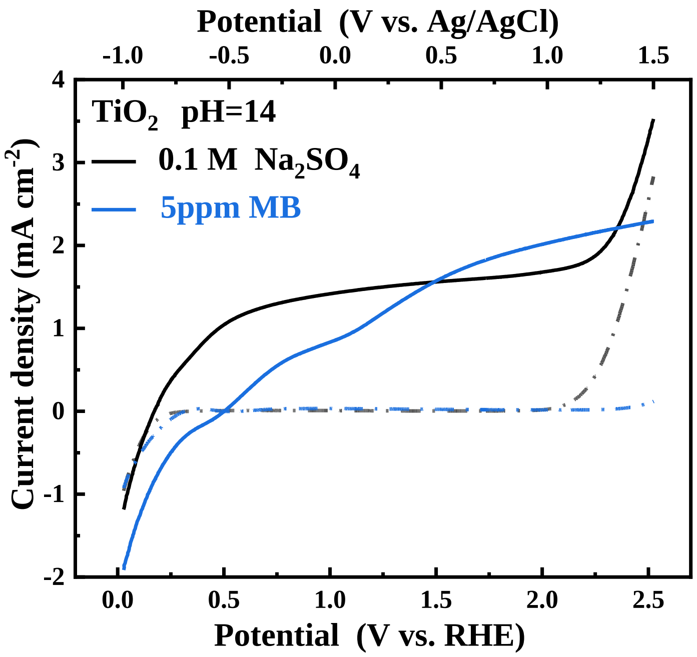

Figure S10. Current density–potential response of the TiO_2 photoanode measured at pH 14 under the illumination of a 365 nm LED.

Table S1. Reaction order α and rate constant lnk of degradation kinetics of methylene blue and methylene blue intermediates under different experimental conditions.

TiO_2 at pH 6	α	logk
Water	2.29	1.66
5ppm MB	2.11	1.98
5ppm MB*	2.07	2.01

TiO_2 at pH 14	α	logk
Water	3.25	1.88
5ppm MB	2.11	1.74
5ppm MB*	3.31	1.62

α-Fe_2O_3 at pH 14	α	logk
Water	3.10	0.45
5ppm MB	2.17	0.35
5ppm MB*	3.21	0.28
